# Sanatoria for Kent

**Published:** 1910-09-10

**Authors:** William J. Howarth

**Affiliations:** County M.O.H., Kent.


### Editor's Letter-Box.

### SANATORIA FOR KENT.

*To the Editor of* The Hospital.

Sir,—Your note on the above subject in the current issue of The Hospital is incorrect. The division of the county to which you refer simply indicates the sanitary districts which would be most suitable for forming combined areas for the purpose of dealing with tuberculous cases, but it is not intended to advise the erection of a sanatorium in each division. The matter is under consideration, and, as you state, it is proposed at an early date to call a meeting of district medical officers to consider the whole subject.

I should be obliged if you would note this correction.

Yours faithfully,

William J. Howarth, County M.O.H., Kent.

THE HOSPITAL. September 10, 1910.

